# Early Complication Rates Are Equivalent Between Isolated Cartilage Restoration and Concomitant Cartilage Restoration and Osteotomy of the Knee

**DOI:** 10.1177/19476035231194769

**Published:** 2023-08-17

**Authors:** Hunter Bohlen, Theofilos Karasavvidis, Deborah Wen, Francis K.L. Wong, Dean Wang

**Affiliations:** 1 Department of Orthopaedic Surgery, University of California—Irvine, Orange, CA, USA; 2 Department of Orthopaedic Surgery, Sengkang General Hospital, Musculoskeletal Sciences ACP, Singhealth-DukeNUS Graduate Medical School, Sengkang General Hospital, Singapore; 3 Department of Biomedical Engineering, University of California—Irvine, Irvine, CA, USA

**Keywords:** cartilage repair, osteotomy, articular cartilage, autologous chondrocyte implantation, cartilage transplantation

## Abstract

**Objective:**

Realignment osteotomy performed concomitantly with cartilage restoration typically requires early restricted weightbearing and can add significant morbidity, potentially leading to an increased risk of early perioperative complications. The purpose of this study was to compare the 30-day complication rates after isolated cartilage restoration (ICR) versus concomitant cartilage restoration and osteotomy (CRO) using the American College of Surgeons National Surgical Quality Improvement Program (ACS-NSQIP) database.

**Design:**

NSQIP registries between 2006 and 2019 were queried using Current Procedural Terminology codes to identify patients undergoing ICR (autologous chondrocyte implantation, osteochondral autograft transfer, or osteochondral allograft transplantation) and CRO (with concomitant high tibial osteotomy, distal femoral osteotomy, and/or tibial tubercle osteotomy). Complications rates between treatment groups were compared using multivariate logistic regression analyses adjusted for sex, age, steroid use, and respiratory status.

**Results:**

A total of 773 ICR and 97 CRO surgical procedures were identified. Mean patient ages were 35.9 years for the ICR group and 31.2 years for the CRO group. Operative time was significantly longer in the CRO group (170.8 min) compared with the ICR group (97.8 min). Multivariate analysis demonstrated no significant differences in rates of PE, VTE, and all-cause readmission between the ICR and CRO groups. No events of wound disruption, SSI and reoperation were found in the CRO group, while the ICR group was characterized by low rates of wound disruption, reoperation, and SSI (<1.1%).

**Conclusions:**

These findings further support concomitant osteotomy with cartilage restoration when appropriate and aid surgeons in the preoperative counseling of patients undergoing cartilage restoration treatment.

## Introduction

Articular cartilage damage represents a common source of knee pain and can be difficult to manage in young patients.^1-3^ Cartilage restoration surgery is often indicated in these patients for focal lesions in order to treat pain, joint dysfunction, and prevent further cartilage deterioration. Multiple cartilage restoration techniques have been successfully described, including autologous chondrocyte implantation (ACI), osteochondral autograft transfer (OAT), and osteochondral allograft transplantation (OCA).^4,5^ The success of cartilage restoration surgery is predicated on a thorough assessment of anatomic factors including mechanical axis alignment, ligamentous stability, and meniscus integrity, as concurrent pathology may indicate the need for additional surgery to maximize the success of the cartilage restoration.^2^

Mechanical axis deviation of the lower limb changes the load-bearing forces on the knee and can exacerbate progression of osteoarthritis and other articular cartilage defects.^6,7^ Knee joint malalignment can be corrected via osteotomy, which aims to restore anatomic alignment and decrease biomechanical loading through the affected compartment.^7,8^ Common periarticular osteotomy procedures include high tibial osteotomy (HTO) and distal femoral osteotomy (DFO) for addressing coronal, sagittal, and rotational malignment, and tibial tubercle osteotomy (TTO) for addressing patellofemoral malalignment.^2,8-11^ Although cartilage restoration procedures and osteotomy have been studied extensively in isolation, limited research exists regarding concomitant cartilage restoration and osteotomy (CRO). A growing number of mostly small case series on CRO surgery have demonstrated improved clinical outcomes and graft survivorship.^12-20^ However, perioperative risks that may be increased with concomitant osteotomy including infection, venous thromboembolism (VTE), fracture, malunion/nonunion, vascular complications, and neurovascular damage.^9,21^ These risks may push surgeons to avoid combined osteotomy when addressing cartilage lesions in patients with malalignment. However, whether concomitant osteotomy increases the rate of these risks in the setting of a cartilage repair procedure is unknown.

To date, there are few studies comparing the complication rates in patients undergoing isolated cartilage restoration (ICR) versus concomitant cartilage restoration and osteotomy (CRO). Therefore, the purpose of this study was to compare the 30-day postoperative complication rates of ICR and CRO surgery using the American College of Surgeons National Surgical Quality Improvement Program (ACS-NSQIP) database.

## Methods

### Data Source

The data for this study were retrospectively collected from the American College of Surgeons National Quality Improvement Program (ACS-NSQIP). The ACS-NSQIP is a prospectively collected, risk-adjusted, outcomes-based program with over 500 participating institutions in the United States. The 2019 version of the database was used, which contains more than 300 variables including preoperative risk factors, intraoperative variables, and 30-day postoperative complications for patients undergoing major surgical procedures. The database is maintained and updated by trained clinical reviewers, who extract patient information from patient interview, medical records, and operative reports through the 30th postoperative day.^22^ The study was conducted according to the Strengthening the Reporting of Observational Studies in Epidemiology (STROBE) guidelines.^23^ Ethical permission for this retrospective database review was done in accordance with the University of California, Irvine Institutional Review Board, who deemed this study to be human subjects exempt.

### Patient Population

Current Procedural Terminology (CPT) codes were utilized to identify eligible patients for this study. First, patients undergoing a knee cartilage restoration procedure between January 1, 2006, and December 31, 2019, with autologous chondrocyte implantation (ACI) (27412), open or arthroscopic osteochondral autograft transfer (OAT) (27416/29866), and open or arthroscopic osteochondral allograft transplantation (OCA) (27415/29867) were identified. This population was then dichotomized into 2 groups: patients undergoing ICR and patients undergoing CRO. Concomitant osteotomy was recorded for high tibial osteotomy (HTO) (27455, 27457), distal femoral osteotomy (DFO) (27448, 27450) and tibial tubercle osteotomy (TTO) (27418) performed during the same surgery as the cartilage procedure.

Patient demographic characteristics including gender, age, body mass index (BMI), and history of comorbidities, including diabetes, smoking, dyspnea, chronic obstructive pulmonary disease (COPD), hypertension (HTN), steroid use, and bleeding disorders were extracted for analysis. Operative time was also collected. Postoperative 30-day complications and outcomes of interest included deep vein thrombosis (DVT), pulmonary embolism (PE), total venous thromboembolism (VTE), wound disruption, surgical site infection (SSI), reoperation, and all-cause readmission. Total VTE was defined as an event of DVT or PE.

### Statistical Analysis

Continuous variables were described with mean ± standard deviation, whereas categorical variables were reported with absolute and relative frequencies. Kolmogorov-Smirnov and Skewness-Kurtosis tests showed nonnormal distributions for variables in both the ICR and CRO groups. In addition, the unequal sample sizes between ICR and CRO groups were recognized. Therefore, the Wilcoxon rank sum test with appropriate corrections to account for unequal sample size and variance was conducted to compare continuous variables;^24^ binary outcomes were compared using the Chi-square or Fisher exact test as appropriate.^25^ Both statistical tests have been shown to be resistant to variations in sample size ratio up to 10;^26-28^ the sample size ratio in this study was 7.97. Multivariate logistic regression analyses were performed to compare complication rates between ICR and CRO. The following variables were decided *a priori* to be included in the multivariate model: cartilage restoration treatment, patient sex, patient age, and operative time. Subgroup analyses were conducted for each cartilage restoration procedure. The threshold for statistical significance was *P* < 0.05. Stata 17 (StataCorp LLC, College Station, TX) was used as statistical software for all analyses.

## Results

### Patient Characteristics

A total of 773 ICR and 97 CRO surgical procedures were identified. The ICR group included 132 ACI, 253 OAT, and 388 OCA procedures, while the CRO consisted of 33 HTO, 5 DFO, and 59 TTO. Table 1 shows the demographic characteristics and preexisting comorbidities of all patients included in the final analysis.

**Table 1.** Patient Demographics.

Variables	Isolated Cartilage Restoration (*n* = 773), *n* (%)	Cartilage Restoration + Osteotomy (*n* = 97), *n* (%)	*P* value
Cartilage procedure			<0.001
ACI	132 (17.1)	31 (32)	
OAT	253 (32.7)	13 (13.4)	
OCA	388 (50.2)	53 (54.6)	
Female	286 (37)	39 (40.2)	0.5
Age (mean, SD)	35.9 (12.7)	31.2 (8.2)	<0.001
BMI (mean, SD)	29.6 (6.8)	30.4 (5.6)	<0.02
Diabetes	21 (2.7)	0	0.4
Smoking	121 (15.6)	11 (11.3)	0.2
Dyspnea	8 (1)	0	0.3
COPD	3 (0.4)	0	0.5
HTN	100 (12.9)	11 (11.3)	0.6
Steroid use	9 (1.2)	0	0.2
Bleeding disorders	3 (0.4)	0	0.5

Mean patient ages were 35.9 years for the ICR group and 31.2 years for the CRO group (*P* < 0.001). The 2 groups were statistically similar in terms of gender, diabetes, smoking, dyspnea, COPD, HTN, steroid use, and bleeding disorders.

ACI = autologous chondrocyte implantation; OAT = osteochondral autograft transfer; OCA = osteochondral allograft transplantation; BMI = body mass index; COPD = chronic obstructive pulmonary disease; HTN = hypertension.

### Outcomes and Complications

Operative time was significantly longer in the CRO group versus the ICR group (mean time: 170.8 vs. 97.8 min, *P* < 0.001). No events of 30-day wound disruption, SSI and reoperation were found in the CRO group, while the ICR group was characterized by low rates of wound disruption (0.26%), reoperation (0.52%), and SSI (1.03%). Multivariate analysis revealed no significant differences between the ICR and CRO groups in terms of PE (0.13% vs. 1.03%), total VTE (0.65% vs. 2.06%), and all-cause readmission (0.65% vs. 1.03%) (Table 2). Details on the complications for each procedure are listed in Table 3.

**Table 2.** 30-Day Postoperative Complications.

Complication	Isolated Cartilage Restoration (*n* = 773)	Cartilage Restoration + Osteotomy (*n* = 97)	Univariate Analysis		Multivariate Analysis	
	*n* (%)	*n* (%)	OR (95% CI)	*P* value	OR (95%CI)	*P* value
PE	1 (0.13)	1 (1.03)	8 (0.5-129.6)	0.1	4.2 (0.2-87.2)	0.3
VTE	5 (0.65)	2 (2.06)	3.2 (0.6-16.9)	0.2	3.6 (0.5-27.5)	0.2
Wound disruption	2 (0.26)	0	1		1	
Reoperation	4 (0.52)	0	1		1	
Readmission	5 (0.65)	1 (1.03)	1.4 (0.2-12.2)	0.7	3.2 (0.2-45.4)	0.4
Surgical site infection	8 (1.03)	0	1		1	

No complications were noted for concomitant ACI or OAT with osteotomy, while all complication rates were <2% for each isolated cartilage repair procedure (ACI, OAT, and OCA) (Table 3).

OR = odds ratio; CI = confidence interval; PE = pulmonary embolism; VTE = venous thromboembolism.

**Table 3.** ACI, OAT, and OCA Complications.

Complication	Isolated OAT (*n* = 253)	Osteotomy + OAT (*n* = 13)	Isolated ACI (*n* = 132)	Osteotomy + ACI (*n* = 31)	Isolated OCA (*n* = 388)	Osteotomy + OCA (*n* = 53)
	*n* (%)	*n* (%)	*n* (%)	*n* (%)	*n* (%)	*n* (%)
PE	0	0	1 (0.76)	0	0	1 (1.89)
VTE	2 (0.79)	0	2 (1.52)	0	1 (0.26)	2 (3.77)
Wound disruption	0	0	0	0	2 (0.52)	0
Reoperation	2 (0.79)	0	1 (0.76)	0	1 (0.26)	0
Readmission	3 (1.4)	0	2 (1.77)	0	0	1 (1.89)
Surgical site infection	5 (1.98)	0	1 (0.76)	0	2 (0.52)	0

ACI = autologous chondrocyte implantation; OAT = osteochondral autograft transfer; OCA = osteochondral allograft transplantation; PE = pulmonary embolism; VTE = venous thromboembolism.

## Discussion

This retrospective ACS-NSQIP database study identified 870 patients who underwent articular cartilage restoration (773 ICR and 97 CRO surgical procedures) and demonstrated no differences in 30-day complications, including PE, VTE, wound complications, infection, readmission, and reoperation, after ICR versus CRO. Overall, low 30-day complication rates were observed in both groups. These data suggest that despite significantly increased operative time for CRO versus ICR (170.8 vs. 97.8 min), concomitant osteotomy can be performed in the setting of cartilage restoration without increasing the risk of perioperative complications.

Although limited research exists examining outcomes and complications of combined cartilage restoration procedures and osteotomy, the findings of this study contribute to a growing body of evidence regarding the safety and efficacy of CRO surgery. A recent matched cohort study of 1,113 patients (402 ACI, 67 ACI + osteotomy, 552 OCA, 92 OCA + osteotomy) reviewed 30-day reoperation rate and all-cause return to operating room after minimum 2-year follow-up.^29^ Reoperation rate was significantly higher after ICR compared with CRO at 2-year follow-up, with a reoperation rate of 68.7% for the isolated ACI group versus 23.9% for ACI plus osteotomy, and a rate of 34.8% for isolated OCA compared with 16.3% for OCA plus osteotomy. Postoperative 30-day complications were infrequent and no statistically significant difference was identified between the 2 groups, with short-term complication rates <3% for infection, DVT, hematoma, sepsis, heterotopic ossification, and knee dislocation.^29^ These results were comparable with what was observed in this study.

Cotter *et al.*^11^ recently reported on risk factors associated with short-term complications for patients undergoing isolated osteotomy of the knee. This study reviewed database data on 1,083 patients undergoing DFO (28%), HTO (25%), or TTO (47%). Statistically significant independent risk factors for failure included age >45 for DFO and HTO (OR 3.1 and 2.3, respectively), and BMI >30 for HTO (OR 2.5). Analysis of all osteotomies identified age >45 (OR 4.1), diabetes (OR 2.2), COPD (OR 5.5), and dependent functional status (OR 3.0) to be associated with higher rates of short-term adverse events. Overall, 30-day complication rates were reported as 22.3% for DFO, 9.9% for HTO, and 4.6% for TTO. This study reflected an older and less healthy patient population than was present in this study, which may explain the higher short-term complication rates that were reported. Both this study and Calcei *et al.*^29^ described cohorts which were overall young and healthy, with average patient age under 36 years, as well as low rates of obesity, diabetes, and tobacco use. While data from these studies suggest that CRO can be performed safely in a young, healthy patient population, this may not be the case in older patients with comorbidities including DM, COPD, or dependent functional status.^11^

Limited literature exists comparing long-term outcomes of CRO versus ICR. Faber *et al.*^30^ recently published a German database series comparing 220 matched patients with medial compartment cartilage lesions undergoing isolated cartilage repair with those undergoing combined cartilage repair and HTO. This study demonstrated improvements in Knee Injury and Osteoarthritis Outcome Score (KOOS), visual analogue scale (VAS), and patient-reported satisfaction scores in the CRO group at 12, 24, and 36 months, with results more pronounced in patients with varus deformity >5°. This study did not discuss complication or reoperation rates.^30^

Several smaller case series demonstrate improved long-term outcomes for CRO surgery with few short-term complications.^1,8,12-20,31^ Haunschild *et al.*^31^ published a series of 24 patients with lateral chondral defects and valgus deformity who underwent concomitant DFO and OCA with an average of 7.13 years follow-up. Only 2 cases were considered failures at final follow-up (both undergoing subsequent OCA surgery); however, a total of 8 patients required reoperation, with 5 returning for hardware removal. Overall, patients achieved significant improvement in post-operative KOOS, Lysholm, and International Knee Documentation Committee (IKDC) outcome scores at final follow-up. Trinh *et al.*^19^ reported on 11 studies including 366 patients who underwent either isolated ACI surgery (78%) or combined ACI and TTO (22%) for patellofemoral cartilage lesions, with a mean follow-up of 4.2 years. In studies comparing ICR to CRO, knee outcome scores were significantly better in the CRO group (*P* < 0.05). In addition, no differences in postoperative complication rates were noted.

The results of this study support that CRO surgery is safe despite increased operative time, with no statistically significant difference in 30-day complication rates between CRO and ICR surgery. These data are congruent with other literature regarding 30-day complication rates after CRO surgery, adding to a growing body of evidence that osteotomy should be considered in the setting of cartilage restoration in young, healthy patients if malalignment exists. These results may not hold for older patients with significant comorbidities, as higher short-term complication rates have been noted after isolated osteotomy in older, less healthy patients.^11^ However, many of these older patients are not candidates for cartilage restoration due to diffuse degenerative disease. Although long-term research is limited given the infrequent nature of CRO surgery, studies available demonstrate that patients with early cartilage injury and malalignment have maintained improvement in knee outcome scores at long-term follow-up. In addition, in a matched cohort of patients with varus deformity and medial cartilage injury, those undergoing CRO surgery had statistically significant improvement in knee outcome scores compared with those undergoing ICR surgery at 3-year follow-up.^30^

Several limitations exist with this study. Inherent in any database study, data are dependent on accurate International Classification of Diseases (ICD) and CPT coding. Any coding inaccuracies by treating physicians contribute to a potential source of error. Complications were only recorded within a 30-day window, and as such, complications and reoperations occurring after 30 days were not captured by this study. Known longer term complications or re-operations, such as need for hardware removal after CRO surgery, would not be elucidated by this study design and must be considered when counseling patients. Several potentially confounding variables were not captured by this database, including prior surgical procedures, chronicity of pathology, cartilage lesion characteristics, degree of mechanical malalignment/correction, and other factors which contribute to surgical decision-making on young patients with articular cartilage injuries. Follow-up studies analyzing these factors may provide further insight into the risk factors for complications in patients undergoing these procedures.

In this study, no differences in 30-day PE, VTE, and all-cause readmission after ICR versus CRO were observed. Complication rates were low overall (<2.1%). These findings further support concomitant osteotomy with cartilage repair when performed in the setting of associated limb or patellofemoral malalignment.
